# Neuronal TCF7L2 in Lateral Habenula Is Involved in Stress-Induced Depression

**DOI:** 10.3390/ijms252212404

**Published:** 2024-11-19

**Authors:** Xincheng Li, Xiaoyu Liu, Jiaxin Liu, Fei Zhou, Yunluo Li, Ye Zhao, Xueyong Yin, Yun Shi, Haishui Shi

**Affiliations:** 1 Neuroscience Research Center, Institute of Medical and Health Science, Hebei Medical University, Shijiazhuang 050017, China; 22031070004@stu.hebmu.edu.cn (X.L.); lxy1195@stu.hebmu.edu.cn (X.L.); 17736103895@163.com (J.L.); 20202407@stu.hebmu.edu.cn (F.Z.); tmm90803@gmail.com (Y.L.); 19501828@hebmu.edu.cn (Y.Z.); 20212063@stu.hebmu.edu.cn (X.Y.); 2 Hebei Key Laboratory of Neurophysiology, Hebei Medical University, Shijiazhuang 050017, China; 3 Hebei Key Laboratory of Early Life Health Promotion, College of Nursing, Hebei Medical University, Shijiazhuang 050031, China

**Keywords:** chronic mild stress, depression, transcription factor 7-like 2, lateral habenula, N-methyl-D-aspartate receptor

## Abstract

Depression is a complex psychiatric disorder that has substantial implications for public health. The lateral habenula (LHb), a vital brain structure involved in mood regulation, and the N-methyl-D-aspartate receptor (NMDAR) within this structure are known to be associated with depressive behaviors. Recent research has identified transcription factor 7-like 2 (TCF7L2) as a crucial transcription factor in the Wnt signaling pathway, influencing diverse neuropsychiatric processes. In this study, we explore the role of TCF7L2 in the LHb and its effect on depressive-like behaviors in mice. By using behavioral tests, AAV-mediated gene knockdown or overexpression, and pharmacological interventions, we investigated the effects of alterations in TCF7L2 expression in the LHb. Our results indicate that TCF7L2 expression is reduced in neurons within the LHb of male ICR mice exposed to chronic mild stress (CMS), and neuron-specific knockdown of TCF7L2 in LHb neurons leads to notable antidepressant activity, as evidenced by reduced immobility time in the tail suspension test (TST) and forced swimming test (FST). Conversely, the overexpression of TCF7L2 in LHb neurons induces depressive behaviors. Furthermore, the administration of the NMDAR agonist NMDA reversed the antidepressant activity of TCF7L2 knockdown, and the NMDAR antagonist memantine alleviated the depressive behaviors induced by TCF7L2 overexpression, indicating the involvement of NMDAR. These findings offer novel insights into the molecular mechanisms of depression, highlighting the potential of TCF7L2 as both a biomarker and a therapeutic target for depression. Exploring the relationship between TCF7L2 signaling and LHb function may lead to innovative therapeutic approaches for alleviating depressive symptoms.

## 1. Introduction

Depression is a complex and multifaceted psychiatric disorder that represents a major public health concern globally, affecting millions of people each year [1]. It has become the leading cause of disability worldwide and is often life-threatening. Furthermore, it is a major contributor to suicide, claiming nearly 800,000 lives globally each year [1,2]. Despite significant advances in understanding the neurobiological underpinnings of depression, the precise mechanisms that drive the onset and persistence of this disorder remain elusive [3,4]. Among the brain structures implicated in depression, the lateral habenula (LHb) has emerged as a critical node in the regulation of mood and emotional responses, acting as a central hub that integrates aversive stimuli and modulates downstream reward-related circuits [5,6,7,8].

The LHb, located in the epithalamus, has been shown to play a pivotal role in processing negative valence signals and the pathophysiology of depression [9,10,11]. This brain region is specifically involved in the inhibition of dopamine and serotonin release, two neurotransmitters that are essential for mood regulation [8]. Aberrant activity within the LHb has been linked to various depressive symptoms, including anhedonia and helplessness [12]. The results of a number of studies have demonstrated that after repeated exposure to stressors, animals predominantly display depressive-like behaviors and hyperactivity of LHb neurons, characterized by increased bursting activity [13,14]; moreover, this hyperactivity in the LHb can be reversed by the use of antidepressant treatments, highlighting its significance in the pathophysiology of depression [12,15,16,17].

Cells within the LHb are predominantly glutamatergic [18]. Functionally, these glutamatergic LHb neurons are heterogeneous in terms of their response to aversive stimuli [19]. A key player in the function of LHb neurons is the N-methyl-D-aspartate receptor (NMDAR), a glutamate receptor that is crucial for synaptic plasticity, neurotransmitter release, and the regulation of neuronal excitability. The NMDAR in the LHb has been implicated in the development of depressive symptoms, particularly through its role in the modulation of excitatory neurotransmission [20]. Increased NMDAR activity in the LHb has been associated with heightened excitability of LHb neurons, leading to the promotion of aversive and depressive behaviors [16,20,21]. Such findings suggest that targeting NMDAR signaling in the LHb may offer a potential therapeutic avenue for alleviating depression.

In parallel, recent research has highlighted the significance of the Wnt signaling pathway in brain function and development, with a particular focus on transcription factor 7-like 2 (TCF7L2), a transcription factor within this pathway [22,23]. TCF7L2 has been recognized as a critical regulator of various neurodevelopmental and neuropsychiatric processes, including neuronal differentiation, synaptic plasticity, and behavioral modulation [24,25,26,27]. Intriguingly, genetic variations in the *Tcf7l2* gene have been linked to an increased risk of type 2 diabetes, a metabolic disorder often co-occurring with depression [24,26,28]. This genetic connection suggests a potential overlap between metabolic and mood-related pathways, further complicating the landscape of depression’s etiology.

The role of TCF7L2 in the LHb, particularly its relationship with NMDAR, remains an area of active investigation. The results of previous studies suggest that the dysregulation of TCF7L2 may contribute to altered neuronal activity and synaptic plasticity, processes that are essential for mood regulation [28,29,30,31]. Given the established roles of both the LHb and TCF7L2 in mood regulation, we wondered whether TCF7L2 within LHb neurons contributes to the manifestation of depressive-like behaviors and, if so, whether this effect is mediated through NMDAR signaling.

In this study, we employed a combination of behavioral tests, AAV-mediated gene manipulation, and pharmacological interventions to examine the effect of TCF7L2 expression in the LHb on depressive-like behaviors in mice. Our results showed that TCF7L2 was downregulated in the CMS mouse LHb neurons. However, LHb neuron-specific knockdown of TCF7L2 leads to notable antidepressant activity, as evidenced by decreased immobility in the tail suspension test (TST) and forced swim test (FST). Conversely, overexpression of TCF7L2 in LHb neurons induces depressive-like behaviors. Furthermore, the administration of the NMDAR agonist NMDA reversed the antidepressant activity of TCF7L2 knockdown, and the NMDAR antagonist memantine alleviated the depressive-like behaviors induced by TCF7L2 overexpression.

These findings support our hypothesis that TCF7L2 in the LHb plays a critical role in regulating depressive-like behaviors, potentially through modulating NMDAR activity. Our study not only deepens our understanding of the neurobiological basis of depression but also highlights the therapeutic potential of targeting TCF7L2 signaling in the LHb.

## 2. Results

### 2.1. The Expression Levels of TCF7L2 Were Decreased in the LHb of Mice Exposed to CMS

**Figure 1:**
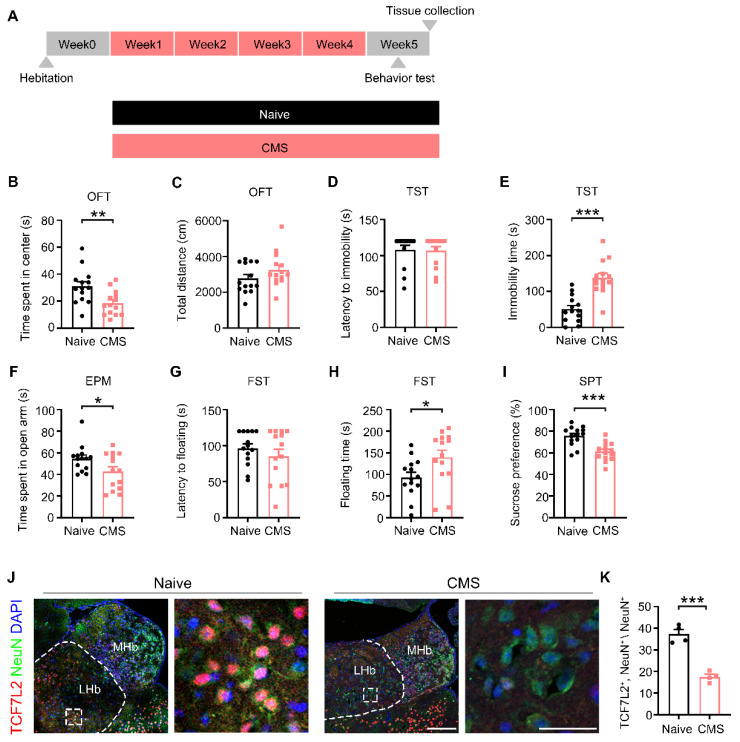
TCF7L2 in LHb neurons was downregulated in CMS mice. (**A**) Timeline of the CMS and behavioral tests, including the OFT, the TST, the EPM, the FST, and the SPT. (**B**) Time spent in the center in the OFT, mean of Naive: 30.98, mean of CMS: 18.46. (**C**) Total distance traveled during the OFT, mean of Naive: 2773, mean of CMS: 3258. (**D**) Latency to the first immobility in the TST, mean of Naive: 108.07, mean of CMS: 106.71. (**E**) Total immobility time in the TST, mean of Naive: 51.2, mean of CMS: 138.1. (**F**) Time spent in the open arms in the EPM, mean of Naive: 54.63, mean of CMS: 42.79. (**G**) Latency to the first floating in the FST, mean of Naive: 96.36, mean of CMS: 85.21. (**H**) Total floating time in the FST, mean of Naive: 93, mean of CMS: 139.9. (**I**) Sucrose preference (%) of SPT, mean of Naive: 75.69, mean of CMS: 61.18, (**J**,**K**) Immunofluorescence staining of TCF7L2 in LHb neurons, mean of Naive: 37.34, mean of CMS: 17.47, (red: TCF7L2, green: NeuN, blue: DAPI, scale bar = 50 μm; n = 4). Comparison between the Naive and CMS groups was conducted using T-tests or Mann–Whitney U-tests. Data are expressed as means ± SEM. n = 14 per group. * *p* < 0.05, ** *p* < 0.01, *** *p* < 0.001, versus the Naive group.

The CMS paradigm (Figure 1A) was used to induce depressive-like behavior in adult male mice. Twenty-four hours after CMS, behavioral tests were performed. In the OFT, compared with the Naive group, the CMS group showed less time spent in the center of the OFT (t_26_ = 3.014, *p* = 0.0057; Figure 1B) with no difference in total distance traveled (t_26_ = 1.431, *p* = 0.1642; Figure 1C). In the EPM, mice in the CMS group spent less time in the open arm (t_26_ = 2.150, *p* = 0.0410; Figure 1F), indicating significant anxiety-like behavior after 4 weeks of CMS.

In the TST, the CMS group showed no difference in latency to the first immobility compared with the Naive group (U = 93, *p* = 0.8388; Figure 1D) but exhibited a longer immobility time in the total 4 min (t_26_ = 0.5471, *p* < 0.0001; Figure 1E). Similarly, in the FST, there was no difference in the latency to the first immobility between the CMS group and the Naive group (t_26_ = 0.9682, *p* = 0.3419; Figure 1G); however, the CMS group showed a longer floating time in the total 4 min (t_26_ = 2.325, *p* = 0.0281; Figure 1H). In the SPT, the sucrose preference rate was lower in the CMS group than in the Naive group (t_26_ = 4.448, *p* = 0.0001; Figure 1I). These results demonstrate characteristic depressive-like phenotypic features: despair-like behavior and anhedonia.

Next, we used fluorescence staining to analyze TCF7L2 expression in neurons in the LHb region. The result showed a significant decrease in TCF7L2 in LHb neurons in the CMS group compared to the Naive group (t_6_ = 8.333, *p* = 0.0002; Figure 1K). These findings suggest that the reduction in TCF7L2 in LHb neurons may play a role in CMS-induced depressive-like behavior in mice.

### 2.2. LHb Neuron-Specific Knockdown of TCF7L2 Caused Antidepressant Activity in Mice

**Figure 2:**
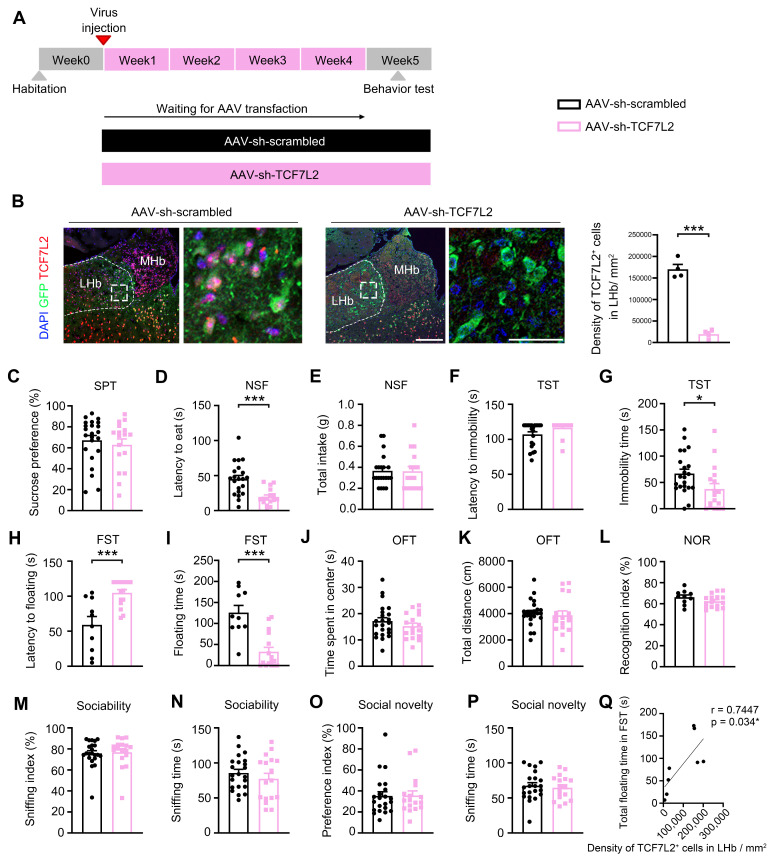
TCF7L2 knockdown in the LHb neurons caused antidepressant activity in mice. (**A**) Schematic of the experimental design of AAV-mediated TCF7L2 knockdown in the LHb neurons of mice. (**B**) Verification of TCF7L2 knockdown efficiency using fluorescence staining, mean of AAV-sh-scrambled: 93, mean of AAV-sh-TCF7L2: 139.9 (red: TCF7L2, green: GFP, blue: DAPI, scale bar = 50 μm). (**C**) Sucrose preference (%) of SPT, mean of AAV-sh-scrambled: 67.24, mean of AAV-sh-TCF7L2: 62.92. (**D**) Latency to eat in the NSF test, mean of AAV-sh-scrambled: 45.1, mean of AAV-sh-TCF7L2: 19.5. (**E**) Total intake of food in the NSF test, mean of AAV-sh-scrambled: 0.3667, mean of AAV-sh-TCF7L2: 0.3625. (**F**) Latency to the first immobility in the TST, mean of AAV-sh-scrambled: 107.2, mean of AAV-sh-TCF7L2: 116.5. (**G**) Total immobility time in the TST, mean of AAV-sh-scrambled: 66.86, mean of AAV-sh-TCF7L2: 37.53. (**H**) Latency to the first floating in the FST, mean of AAV-sh-scrambled: 59, mean of AAV-sh-TCF7L2: 104.8. (**I**) Total floating time in the FST, mean of AAV-sh-scrambled: 125.9, mean of AAV-sh-TCF7L2: 32.81. (**J**) Time spent in the center in the OFT, mean of AAV-sh-scrambled: 17.17, mean of AAV-sh-TCF7L2: 15.25. (**K**) Total distance traveled during the OFT, mean of AAV-sh-scrambled: 4039, mean of AAV-sh-TCF7L2: 3870. (**L**) Recognition index of NOR test, mean of AAV-sh-scrambled: 66.21, mean of AAV-sh-TCF7L2: 62.33. (**M**) Sniffing index in trial 1 of the three-chamber SIT, mean of AAV-sh-scrambled: 75.78, mean of AAV-sh-TCF7L2: 76.87. (**N**) Total sniffing time in trial 1 of the three-chamber SIT, mean of AAV-sh-scrambled: 85.82, mean of AAV-sh-TCF7L2: 77.41. (**O**) Preference index in trial 2 of the three-chamber SIT, mean of AAV-sh-scrambled: 35.31, mean of AAV-sh-TCF7L2: 35.61. (**P**) Total sniffing time in trial 2 of the three-chamber SIT, mean of AAV-sh-scrambled: 67.45, mean of AAV-sh-TCF7L2: 64.59. (**Q**) Analysis of the correlation between the total floating time in FST and density of TCF7L2^+^ cells in LHb/mm^2^. Comparison between the AAV-sh-Scrambled and AAV-sh-TCF7L2 groups was conducted using the *T*-test or Mann–Whitney U-test. Data are expressed as means ± SEM. n = 10–23 per group. * *p* < 0.05, *** *p* < 0.001, versus the AAV-sh-Scrambled group.

To examine the role of TCF7L2 in depressive-like behavior, we used neuron-specific AAV to knock down TCF7L2 in LHb neurons (Figure 2A). Fluorescence staining confirmed the injection site and the effectiveness of the knockdown (Figure 2B). In the SPT, no significant difference in preference index was observed between the two groups of mice (t_38_ = 0.6080, *p* = 0.5468; Figure 2C). In the NSF test, the feeding latency of mice in the AAV-sh-TCF7L2 group was significantly shortened (t_35_ = 0.4012, *p* = 0.0003; Figure 2D), with no difference in total food intake (t_38_ = 0.0764, *p* = 0.9395; Figure 2E). In the TST, there was no significant difference in the latency to the first immobility between the two groups (t_37_ = 0.1.986, *p* = 0.0544; Figure 2F); however, the AAV-sh-TCF7L2 group showed a significantly shorter immobility time (t_37_ = 2.224, *p* = 0.0323; Figure 2G). In the FST, the AAV-sh-TCF7L2 group showed a significantly longer latency to the first float (t_24_ = 4.150, *p* = 0.0004; Figure 2H) and a significantly shorter total floating time compared to the AAV-sh-scrambled group (t_24_ = 4.931, *p* < 0.001; Figure 2I). These results indicate that LHb neuron-specific knockdown of TCF7L2 produces antidepressant activity in mice.

We then evaluated the effects of LHb neuron-specific knockdown of TCF7L2 on anxiety, learning, memory, and social behavior. In the OFT, there were no significant differences in the time spent in the center area (t_38_ = 1.049, *p* = 0.3006; Figure 2J) and the total distance traveled between the two groups (t_38_ = 0.4469, *p* = 0.6575; Figure 2K). In the NOR, there were no differences in recognition indices after training between the two groups (t_24_ = 1.508, *p* = 0.1447; Figure 2L). In the SIT, Trial 1 results showed no statistical differences in sociability (U = 163, *p* = 0.5055; Figure 2M) and sniffing time between the AAV-sh-TCF7L2 group and AAV-sh-scrambled group (t_37_ = 0.9348, *p* = 0.3559; Figure 2N). The results of Trial 2 also showed no statistical differences in social novelty preference (U = 180, *p* = 0.8556; Figure 2O) and sniffing time between the two groups (t_37_ = 0.4595, *p* = 0.6486; Figure 2P). These results demonstrate that LHb neuron-specific knockdown of TCF7L2 had no effects on anxiety, learning, memory, sociability, or social novelty preference in the mice. We further analyzed the correlation between the total floating time in FST and the density of TCF7L2+ cells in LHb/mm^2^ between AAV-sh-scrambled and AAV-sh-TCF7L2 mice. Our findings revealed a significantly positive correlation (r = 0.7447, *p* = 0.034; Figure 2Q). 

### 2.3. LHb Neuron-Specific Overexpression of TCF7L2 Led to Depressive Behaviors in Mice

**Figure 3:**
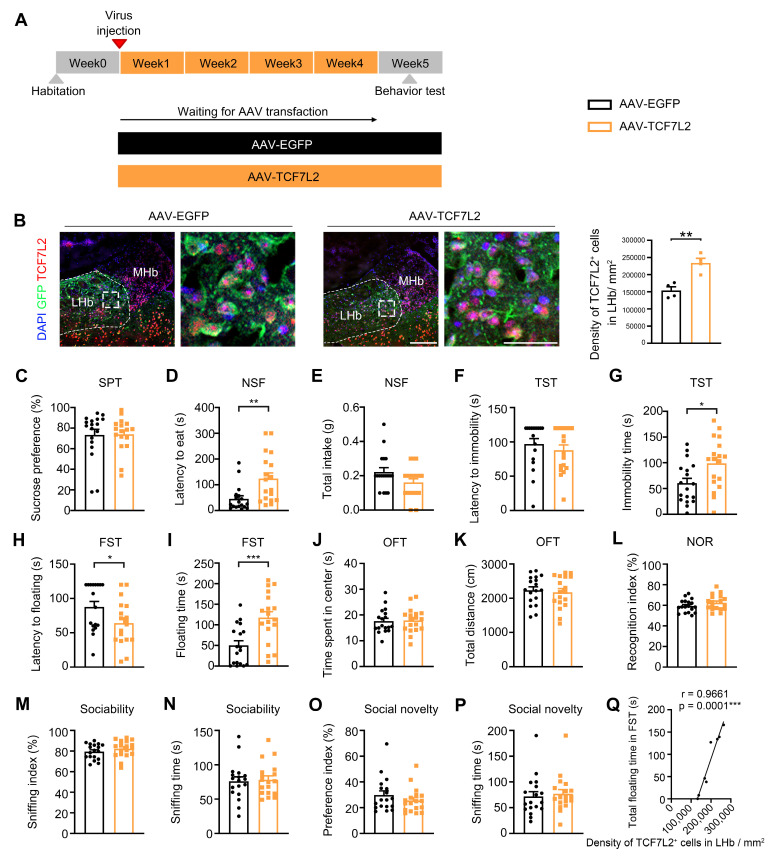
TCF7L2 overexpression in the LHb neurons led to depressive-like behavior in mice. (**A**) Schematic of the experimental design of AAV-mediated TCF7L2 overexpression in the LHb neurons of mice. (**B**) Verification of AAV injection point using fluorescence staining, mean of AAV-EGFP: 67.45, mean of AAV-TCF7L2: 64.59 (red: TCF7L2, green: GFP, blue: DAPI, scale bar = 50 μm). (**C**) Sucrose preference (%) of SPT, mean of AAV-EGFP: 73.5, mean of AAV-TCF7L2: 74. (**D**) Latency to eat in the NSF test, mean of AAV-EGFP: 45.33, mean of AAV-TCF7L2: 124.27. (**E**) Total intake of food in the NSF test, mean of AAV-EGFP: 0.22, mean of AAV-TCF7L2: 0.16. (**F**) Latency to the first immobility in the TST, mean of AAV-EGFP: 97, mean of AAV-TCF7L2:88.06. (**G**) Total immobility time in the TST, mean of AAV-EGFP: 60.39, mean of AAV-TCF7L2: 98.89. (**H**) Latency to the first floating in the FST, mean of AAV-EGFP: 87.67, mean of AAV-TCF7L2: 64.11. (**I**) Total floating time in the FST, mean of AAV-EGFP: 50.06, mean of AAV-TCF7L2: 117.4. (**J**) Time spent in the center in the OFT, mean of AAV-EGFP: 17.61, mean of AAV-TCF7L2: 17.9. (**K**) Total distance traveled during the OFT, mean of AAV-EGFP: 2235, mean of AAV-TCF7L2: 2147. (**L**) Recognition index of NOR test, mean of AAV-EGFP: 59.34, mean of AAV-TCF7L2: 62.5. (**M**) Sniffing index in trial 1 of the three-chamber SIT, mean of AAV-EGFP: 79.49, mean of AAV-TCF7L2: 82.19. (**N**) Total sniffing time in trial 1 of the three-chamber SIT, mean of AAV-EGFP: 75.94, mean of AAV-TCF7L2: 78.22. (**O**) Preference index in trial 2 of the three-chamber SIT, mean of AAV-EGFP: 29.86, mean of AAV-TCF7L2: 25.55. (**P**) Total sniffing time in trial 2 of the three-chamber SIT, mean of AAV-EGFP: 71.94, mean of AAV-TCF7L2: 76.83. Comparison between the AAV-EGFP and AAV-TCF7L2 groups was conducted using *T*-test or Mann–Whitney U-test. (**Q**) Analysis of the correlation between the total floating time in FST and density of TCF7L2^+^ cells in LHb/mm^2^. Data are expressed as means ± SEM. n = 18 per group. * *p* < 0.05, ** *p* < 0.01, *** *p* < 0.001, versus the AAV-sh-Scrambled group.

To further explore the role of TCF7L2 in depressive-like behavior in mice, we injected AAV to overexpress TCF7L2 in the LHb of the mice and performed a series of behavioral tests (Figure 3A). We first verified the injection point of the AAV virus (Figure 3B), confirming that TCF7L2 was indeed overexpressed in LHb neurons. In the SPT, no significant differences were observed between the AAV-EGFP group and the AAV-TCF7L2 group (U = 136, *p* = 0.5905; Figure 3C). In the NSF test, the feeding latency of mice in the AAV-TCF7L2 group significantly increased (U = 62.5, *p* = 0.0012; Figure 3D), with no difference in total food intake (U = 115, *p* = 0.1198; Figure 3E). In the TST, there was no significant difference in the latency to the first immobility between the two groups (t_34_ = 0.8091, *p* = 0.4241; Figure 3F); however, the AAV-TCF7L2 group showed a significantly longer immobility time (t_34_ = 2.589, *p* = 0.0140; Figure 3G). In the FST, the AAV-TCF7L2 group showed a significantly shorter latency to the first float (t_34_ = 2.108, *p* = 0.0425; Figure 3H) and a significantly longer total floating time compared to the AAV-EGFP group (t_34_ = 3.696, *p* = 0.0008; Figure 3I). These results indicate that LHb neuron-specific overexpression of TCF7L2 leads to depressive-like behavior in mice.

Next, we evaluated the effects of LHb neuron-specific overexpression of TCF7L2 on anxiety, learning, memory, and social behavior. In the OFT, there were no significant differences in the time spent in the center area (t_34_ = 0.1843, *p* = 0.8549; Figure 3J) and the total distance traveled between the two groups (t_34_ = 0.4095, *p* = 0.6847; Figure 3K). In the NOR, there were no differences in recognition indices after training between the two groups (t_34_ = 1.387, *p* = 0.1743; Figure 3L). In the SIT, Trial 1 results showed no statistical differences in sociability (t_34_ = 1.042, *p* = 0.3050; Figure 3M) and sniffing time between the AAV-TCF7L2 group and AAV-EGFP group (t_34_ = 0.2636, *p* = 0.7937; Figure 3N). Trial 2 results also showed no statistical differences in social novelty preference (t_34_ = 1.109, *p* = 0.2753; Figure 3O) and sniffing time between the two groups (t_34_ = 0.3921, *p* = 0.6974; Figure 3P). These results demonstrate that the LHb neuron-specific overexpression of TCF7L2 had no effects on anxiety, learning, memory, sociability, or social novelty preference in the mice. We further analyzed the correlation between the total floating time in FST and the density of TCF7L2^+^ cells in LHb/mm^2^ between AAV-EGFP and AAV-TCF7L2 mice. The results showed a significantly positive correlation (r = 0.9661, *p* = 0.0001; Figure 3Q), which aligns with the findings in the TCF7L2 knockdown. This accentuated our findings that depressive-like behavior is directly linked to TCF7L2 expression in the LHb.

### 2.4. Intervention Targeting NMDAR Activity Prevented the Effects of Altered TCF7L2 Expression in LHb Neurons on Depressive-like Behaviors in Mice

It is recognized that NMDARs in the LHb can mediate depressive-like behaviors [20,21]. We investigated whether NMDARs are involved in the process by which altered expression of TCF7L2 in neurons affects depressive-like behavior in mice.

**Figure 4:**
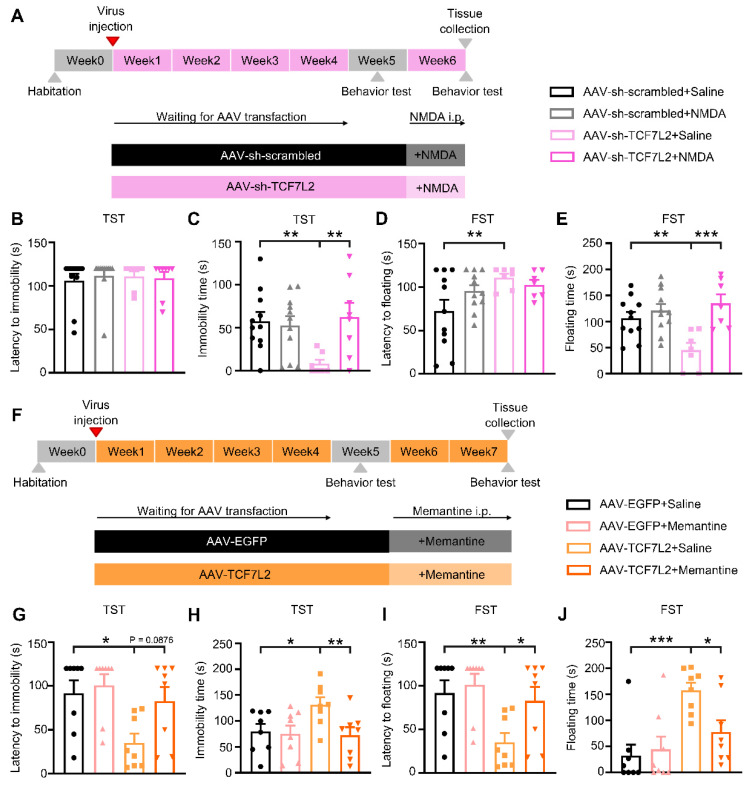
NMDAR was involved in TCF7L2-mediated depressive-like behavior. (**A**) Experimental timeline for NMDAR agonist-NMDA administration and behavioral tests. (**B**) Effects of NMDA administration on latency to the first immobility in the TST with LHb neurons special TCF7L2 knockdown, mean of AAV-sh-scrambled + Saline: 106, mean of AAV-sh-scrambled + NMDA: 111.9, mean of AAV-sh-TCF7L2 + Saline: 111.1, mean of AAV-sh-TCF7L2 + NMDA: 108.8. (**C**) Total immobility time in the TST, mean of AAV-sh-scrambled + Saline: 57.75, mean of AAV-sh-scrambled + NMDA: 52.45, mean of AAV-sh-TCF7L2 + Saline: 8.143, mean of AAV-sh-TCF7L2 + NMDA: 62.63. (**D**) Effects of NMDA administration on latency to the first floating in the FST, mean of AAV-sh-scrambled + Saline: 72.36, mean of AAV-sh-scrambled + NMDA: 95.45, mean of AAV-sh-TCF7L2 + Saline: 110.9, mean of AAV-sh-TCF7L2 + NMDA: 102.6. (**E**) Total floating time in the FST, mean of AAV-sh-scrambled + Saline: 106.1, mean of AAV-sh-scrambled + NMDA: 120.7, mean of AAV-sh-TCF7L2 + Saline: 45, mean of AAV-sh-TCF7L2 + NMDA: 134.7. (**F**) Experimental timeline for NMDAR antagonist-memantine administration and behavioral tests. (**G**) Effects of memantine administration on latency to the first immobility in the TST with LHb neurons special TCF7L2 overexpression, mean of AAV-EGFP + Saline: 85.25, mean of AAV-EGFP + Memantine: 88.25, mean of AAV-TCF7L2 + Saline: 50.75, mean of AAV-TCF7L2 + Memantine: 80.05. (**H**) Total immobility time in the TST, mean of AAV-EGFP + Saline: 79.75, mean of AAV-EGFP + Memantine: 75.38, mean of AAV-TCF7L2 + Saline: 131.5, mean of AAV-TCF7L2 + Memantine: 72.38. (**I**) Effects of memantine administration on latency to the first floating in the FST, mean of AAV-EGFP + Saline: 91.5, mean of AAV-EGFP + Memantine: 100.8, mean of AAV-TCF7L2 + Saline: 35, mean of AAV-TCF7L2 + Memantine: 82.5. (**J**) Total floating time in the FST, mean of AAV-EGFP + Saline: 32.25, mean of AAV-EGFP + Memantine: 44.63, mean of AAV-TCF7L2 + Saline: 157.5, mean of AAV-TCF7L2 + Memantine: 77.88. Comparisons between groups were conducted using one-way ANOVA. Data are expressed as means ± SEM. n = 7–11 per group. * *p* < 0.05, ** *p* < 0.01, *** *p* < 0.001.

First, we used a sub-toxic dose of the NMDAR agonist NMDA (75 mg/kg) [32] and injected it into the mice intraperitoneally. After thirty minutes, we conducted TST and FST tests (Figure 4A). As shown in Figure 4B, there were no significant differences in the latency to the first immobility among the four groups (F_3, 33_ = 0.1425, *p* = 0.9338). There were no significant differences in the immobility time between the AAV-sh-scrambled + NMDA and AAV-sh-scrambled + Saline groups (t_33_ = 0.3352, *p* = 0.7396), indicating that NMDA injection alone did not affect the immobility time in the TST. However, the immobility time in the AAV-sh-TCF7L2 + NMDA group was significantly shorter compared to that in the AAV-sh-TCF7L2 + Saline group (t_33_ = 3.009, *p* = 0.0050) (F_3, 33_ = 3.821, *p* = 0.0187; Figure 4C). In the FST, there were no significant differences in floating time between the AAV-sh-scrambled + NMDA and AAV-sh-scrambled + Saline groups (t_32_ = 1.902, *p* = 0.0662), indicating that NMDA injection alone did not affect floating time in the FST (F_3, 32_ = 3.314, *p* = 0.0390; Figure 4D). However, the floating time in the AAV-sh-TCF7L2 + NMDA group was significantly shorter compared to that in the AAV-sh-TCF7L2 + Saline group (t_32_ = 4.114, *p* = 0.0003) (F_3, 32_ = 6.828, *p* = 0.0011; Figure 4E). These results suggest that NMDAR activation can eliminate the antidepressant activity of LHb-specific TCF7L2 knockdown in mice.

Next, we administered a sub-toxic dose of the NMDAR antagonist memantine (15 mg/kg) to the mice via intraperitoneal injection once daily for two weeks, as this dosage has been shown to have no significant effect on depressive-like behavior [33]. After two weeks, we conducted TST and FST tests (Figure 4F). As shown in Figure 4G, there were no significant differences in the latency to the first immobility (*p* = 0.8596) or immobility time (t_28_ = 0.2066, *p* = 0.8378) between the AAV-EGFP + Memantine and AAV-EGFP + Saline groups, indicating that memantine injection alone did not affect the immobility time in the TST. In addition, there was a significant reduction in the latency to the first immobility between the AAV-EGFP + Saline and AAV-TCF7L2 + Saline groups (t_28_ = 2.053, *p* = 0.0495), and the AAV-TCF7L2 + Memantine group showed an increasing trend compared with the AAV-TCF7L2 + Saline group (t_28_ = 1.770, *p* = 0.0876). The immobility time in the AAV-TCF7L2 + Memantine group was significantly shorter compared to that in the AAV-TCF7L2 + Saline group (t_28_ = 2.792, *p* = 0.0093, Figure 4H).

In the FST, there were no significant differences in latency to the first float (t_28_ = 0.4781, *p* = 0.6363) or floating time (t_28_ =0.4162, *p* = 0.6804) between the AAV-EGFP + Memantine and AAV-EGFP + Saline groups, indicating that memantine injection alone did not affect latency to the first float or floating time in the FST. However, the AAV-TCF7L2 + Memantine group showed shorter latency to the first float than the AAV-TCF7L2 + Saline group (t_28_ = 2.455, *p* = 0.0206, Figure 4I), and the floating time in the AAV-TCF7L2 + Memantine group was significantly shorter compared to that in the AAV-TCF7L2 + Saline group (t_28_ = 2.678, *p* = 0.0122, Figure 4J). These results imply that the influence of modified TCF7L2 expression in LHb neurons on depressive-like behavior in mice is accomplished via the NMDAR.

## 3. Discussion

The results of our study elucidate the critical role of TCF7L2 in the LHb in modulating depressive-like behaviors in mice, providing novel insights into the molecular mechanisms underlying depression. By integrating genetic and pharmacological approaches, we have demonstrated how TCF7L2 expression affects the depressive behavior of mice and identified potential pathways involved.

The CMS paradigm, widely used to model depression in rodents [34], led to a significant reduction in TCF7L2 in the LHb neurons of the mice. The LHb is crucial for encoding negative reward signals and processing aversive stimuli. The results of previous studies have shown that hyperactivity in the LHb is associated with depressive symptoms [8,35,36]. Our findings demonstrate that mice in the CMS group showed a TCF7L2 reduction along with anhedonia and helpless behavior, suggesting that TCF7L2 downregulation may contribute to the occurrence of depressive behaviors. These findings support previous research, indicating that hyperactivity of the LHb may cause greater negative emotional states [16].

Using neuron-specific AAVs to knock down TCF7L2 in LHb neurons, we observed significant antidepressant activity, evidenced by reduced immobility in the TST and FST. These findings are in line with studies showing that modulating LHb activity can alter depressive behaviors. Conversely, overexpressing TCF7L2 in LHb neurons induced significant depressive-like behaviors, including increased immobility in the TST and FST and prolonged feeding latency in NSF. These findings reinforce the notion that TCF7L2 levels in the LHb are critically linked to mood regulation. Notably, the knockdown or overexpression of TCF7L2 did not affect anxiety, learning, memory, or social behaviors, indicating a specific role for TCF7L2 in depression. This specificity highlights the therapeutic potential of targeting TCF7L2, as interventions could potentially mitigate depressive symptoms without broad behavioral side effects.

NMDARs are heterotetramers containing various subunits, including two obligatory GluN1 subunits with eight splice variants, regulatory subunits GluN2 (GluN2A-D) and GluN3 (GluN3A-B) [37]. NMDARs in the LHb are known to be involved in synaptic plasticity and excitatory neurotransmission, both of which are critical for the pathophysiology of depression [21,38]. Therefore, we questioned whether NMDARs mediate the effects of TCF7L2 on depressive-like behaviors in mice. Studies have shown that both NMDA and memantine can cross the blood–brain barrier [39,40]. Given the convenience of clinical application, we chose intraperitoneal injection over local administration in the LHb. Our results showed that the administration of the NMDAR agonist NMDA reversed the antidepressant activity of TCF7L2 knockdown; in comparison, the NMDAR antagonist memantine mitigated the depressive-like behaviors induced by TCF7L2 overexpression. These findings suggest that TCF7L2 may regulate depressive behaviors by modulating the activity of NMDAR, aligning with findings that NMDAR antagonists such as ketamine have rapid antidepressant effects.

The C-terminal domains of NMDA receptor subunits, especially GluN2A and GluN2B, are relatively large and are thought to accommodate nearly all of the phosphorylated amino acids identified to date [41,42]. The results of previous studies have confirmed that phosphorylation of GluN2B at Ser1303 is closely associated with depressive-like behaviors in mice [43]. Moreover, Ca^2+^/calmodulin-dependent protein kinase type II (CaMKII), which mediates the phosphorylation of GluN2B at Ser1303 [44], is a predicted downstream target of TCF7L2. It is likely that TCF7L2 regulates depressive-like behaviors in mice by modulating the phosphorylation of the GluN2B subunit of NMDAR. Investigating the relationship between TCF7L2, CaMKII, and the phosphorylation of GluN2B at Ser1303 will be a key focus of our future research.

We observed a reduction in TCF7L2 expression in neurons within the LHb of CMS mice. Interestingly, the knockdown of TCF7L2 in the LHb neurons resulted in significant antidepressant activity, whereas overexpression of TCF7L2 induced depressive-like behaviors in mice. We hypothesized that the contrasting results may be due to differences in the transcription initiation complex, which were formed by TCF7L2 under the conditions of CMS or LHb neuron-specific TCF7L2 knockdown [45,46]. The next step in our research may involve performing mass spectrometry on different transcription initiation complexes formed by TCF7L2 to analyze differential proteins, which will help validate our hypothesis.

The identification of TCF7L2 as a key modulator of depressive-like behaviors in the LHb offers new therapeutic avenues for depression. Targeting TCF7L2 or its downstream signaling pathways could be a novel strategy for treating depression. Moreover, the interaction between TCF7L2 and NMDARs suggests that combined therapies targeting both TCF7L2 expression and NMDAR activity might offer synergistic benefits. Such therapies could potentially address treatment-resistant depression, a significant clinical challenge.

However, our research group has not yet conducted relevant experiments from the perspective of the neural circuit mechanism, and the impact of TCF7L2 expression changes on the activity of LHb neurons and related mechanisms remains unknown. Therefore, the authors of future studies will need to combine neural circuit methods and transcriptomics or proteomics to conduct detailed investigations on the significant functions of TCF7L2 in LHb neurons. In conclusion, the results of our study establish a pivotal role for TCF7L2 in the LHb in regulating depressive behaviors in mice. By demonstrating the antidepressant activity of TCF7L2 knockdown and the pro-depressive effects of its overexpression, we provide compelling evidence for TCF7L2 as a therapeutic target. Furthermore, our findings on the involvement of NMDARs offer insights into the molecular mechanisms through which TCF7L2 influences mood regulation. These discoveries pave the way for future research and the development of novel therapeutic strategies for depression.

## 4. Materials and Methods

### 4.1. Animals

Male ICR mice (6–7 weeks old on arrival) were obtained from Beijing Vital River Laboratory Animal Technology Co., Ltd. (Beijing, China). Mice were under a constant temperature (22 ± 2 °C) with a controlled 12:12 h light/dark cycle (light on at 8:00 p.m. and light off at 8:00 a.m.) and humidity conditions (50–60%). During this period, all animals had free access to food and water. Behavioral tests were performed during the dark phase. All experiments were reviewed and approved by the Committee on Animal Care and Use of Hebei Medical University, and followed the guidelines of the National Institutes of Health Guide for the Care and Use of Laboratory Animals.

### 4.2. Chronic Mild Stress (CMS)

After 7 days of acclimation, ICR mice were randomly divided into Naive and CMS groups. The model of depression was established as previously described [47]. The animals received two or three different stressors each day, administered on a random schedule, for 4 consecutive weeks. The stresses included damp bedding for 24 h, crowding for 24 h, cage tilting at a 45° angle for 24 h, tail clamping for 1 min, cold stimulation for 10 min, empty cage for 24 h, light/dark cycle reversal for 24 h, food deprivation for 12 h, restraint for 4 h, and water deprivation for 12 h. Naive mice were kept in their home cages under normal conditions and were not subjected to stressors.

### 4.3. Behavioral Tests

#### 4.3.1. Open Field Test (OFT)

To detect the anxiety-like behavior of mice, the OFT was performed based on previous descriptions [48]. In the arena (40 × 40 × 40 cm^3^), the test site (40 × 40 cm^2^) was divided into a central area (20 × 20 cm^2^) and a peripheral area. The mice were free to move and were monitored by a monitor for 5 min. The illumination for this trial was provided by the camera’s infrared light source. The mice’s time spent at the center and the total distance traveled were recorded and analyzed by SMART software (v3.0.02). The reduction in time spent in the central area and the shortening of travel distance are commonly utilized as indicators of anxiety-like behavior.

#### 4.3.2. Elevated Plus Maze (EPM)

The EPM consisted of two open arms and two closed arms that were 50 cm above the floor. A surveillance camera dedicated to behavioral tests was placed above the EPM. Before each experiment, the maze was wiped with 75% alcohol to remove the effects of the previous mouse [48]. Mice were acclimatized to the environment (white light, ~50 lux) for half an hour before the experiment. Mice were placed in the center of the maze and allowed to explore for 5 min and monitored for 5 min with SMART software (V3.0.06). The time spent in the open arms was recorded to assess anxiety-like behavior, and shortened time spent in the open arms is an indication of anxiety-like behavior.

#### 4.3.3. Forced Swimming Test (FST)

Mice were placed in a transparent cylinder, 10 cm in diameter and 35 cm in height, filled with water to a depth of 20 cm (23–25 °C), and allowed to swim for 6 min. The latency to the first floating and total floating time during the last 4 min were recorded. At the end of the FST, the mice were removed from the water, dried with a towel, and returned to their home cages. The latency to the first floating and total floating time were used to evaluate the depressive-like behavior of mice. A shorter latency and longer total immobility time indicate depressive-like behavior.

#### 4.3.4. Tail Suspension Test (TST)

The TST was used to evaluate the depressive-like behavior of mice. A tape was affixed to the test mouse’s tail 2 cm from the tip and the mouse was suspended from a horizontal bar at a height of 30 cm. The mouse was suspended for 6 min and video recordings of the test were quantified by an observer blinded to experimental condition. The latency to the first immobility in the first 2 min and the last 4 min spent in an immobile posture was measured. A shorter latency and longer total immobility time indicate depressive-like behavior.

#### 4.3.5. Sucrose Preference Test (SPT)

The SPT was used to assess stress-induced anhedonia responses [47]. Two bottles of sterile drinking water were placed in a cage and left there for 24 h. During the test, replace one of the bottles with 1% (*w*/*v*) sucrose solution and record the initial weight of both bottles. The filling position was changed every 6 h to prevent positional preference, and the final weight was recorded after 24 h. The sum of the difference before and after sucrose solution and sterile drinking water is considered as the total water intake. The difference before and after the sucrose solution is considered as the sucrose solution intake, and sucrose preference (%) = (sucrose solution intake/total water intake) × 100%. A reduced preference for sucrose solution indicates depressive-like behavior.

#### 4.3.6. Novelty-Suppressed Feeding (NSF) Test

The NSF test serves as a measure of depressive-like behavior in mice [49]. The day after being deprived of food, the mice were placed in the corner of a test box (40 × 40 × 40 cm^3^) in which a single pellet of food had been placed in the central region. The mouse was allowed 5 min to eat the pellet. Latency to feeding was recorded as 5 min if the mouse did not eat, otherwise it was recorded as the time of the first bite within 5 min. Then, the mouse was put back into its home cage and its total food intake was measured for 10 min. A longer latency to eat and lower food intake indicates the presence of depressive-like behavior.

#### 4.3.7. Novel Object Recognition Test (NOR)

The novel object recognition test evaluates memory in mice [50]. Mice were placed near the center of the front wall, facing away from two identical objects in an arena (40 cm × 40 cm × 40 cm) for 5 min. The objects were identical in color, size, and shape, and were sufficiently heavy to prevent the animals from moving them. This initial interaction (habituation phase) was designed to ensure that the animals exhibited no inherent preferences or aversions to either object, and that both objects were explored equally for comparable durations. To evaluate short-term memory, the mice were reintroduced into the arena 30 min after the habituation phase, where they were presented with two objects: one familiar and one novel, the latter differing in both shape and size from the familiar object. Exploration was defined as the act of directing the nose toward the object at a distance of less than 2 cm, or physically touching the object with the nose or mouth. Each session was videotaped for 5 min and subsequently analyzed. The recognition index was calculated as the proportion of time spent exploring the novel object (N) relative to the total exploration time of both the novel (N) and familiar (F) objects, using the formula: N/(N + F) × 100%. The relatively low recognition index indicates that the mice have difficulties in learning and memory.

#### 4.3.8. Social Interaction Test (SIT)

The three-chamber SIT was performed according to the previously described protocol [51]. The test was conducted in a black box consisting of three chambers (length: 33 cm; width: 22 cm; height: 30 cm) with attached doors. The test consisted of two trials that were used to test the sociability and the social novelty of mice, respectively. Prior to the test, the subject mice were placed into the central chamber for 10 min for acclimatization and were allowed to freely explore three chambers. After acclimatization, the mice were placed back into the central chamber while the door was covered with cardboard. An empty metal cage (E) was randomly placed in one side chamber as the nonsocial novel object, and another identical cage containing an unfamiliar stimulus mouse (S1) was placed in another side chamber. Afterward, the cardboard was removed and the subject mice were allowed to freely explore and interact with E or S1 for 10 min (trial 1). The time of mice sniffing E or S1 (t_E_, t_S1_) was recorded. After trial 1, the subject mice were placed back into the central chamber with the door covered by cardboard. Another unfamiliar stimulus mouse (S2) was placed into the previously empty metal cage as the social novelty stimulus, while the S1 was already familiar to the subject mice. The cardboard was then removed and the subject mice were again allowed to freely explore and interact with S2 or S1 for 10 min (trial 2). The time of mice sniffing S2 or S1 (t_S2_, t_S1_) was recorded. The unfamiliar stimulus mice were of the same strain, sex, and age as the subject mice. Sniffing time was defined as the time spent by the subject mice interacting with E, S1, or S2 (e.g., sniffing, touching the nose, head, or forelimbs toward the metal cage). After each session, the box and metal cages were cleaned with 75% alcohol to eliminate the residual odor. The preference index (PI) of sociability and social novelty were calculated as follows: PI_sociability_ = (t_S1_ − t_E_)/(t_S1_ + t_E_); PI_social novelty_ = (t_S2_ − t_S1_)/(t_S2_ + t_S1_). A relatively low sniffing index or preference index indicates that the mice have difficulties in sociability or social novelty preference.

### 4.4. Viral Vector Construction and Injection

Adeno-associated viruses (AAVs) purchased from Genechem Co., Ltd. (Shanghai, China) were used to manipulate TCF7L2 (NM_001142918) expression specifically in LHb neurons. AAV vectors (AAV9-hSyn-EGFP-MIR155(MCS)-WPRE-SV40-PolyA) carrying shRNA targeting TCF7L2 (AAV-sh-TCF7L2) or a scrambled control (AAV-sh-scrambled) were injected into the LHb. For overexpression studies, AAV vectors (AAV9-hSyn-MCS-EGFP-3FLAG-SV40 PolyA) carrying TCF7L2 (AAV-TCF7L2) or an empty vector (AAV-EGFP) were used.

Mice were deeply anesthetized by 1% sodium pentobarbital (100 mg/kg, Sigma, St. Louis, MO, USA) and placed in a stereotactic frame (RWD Instruments). The virus was bilaterally injected into the LHb (0.1 μL; anterior–posterior: −1.82 mm from bregma, medial–lateral: ±0.41 mm, dorsal–ventral: −4.20 mm from the dura) using a pulled glass capillary with a pressure microinjector (Picospritzer III, Parker, Cleveland, OH, USA) at a rate of 0.1 μL/min. The injection needle was withdrawn 10 min after the end of the injection. After surgery, mice recovered from anesthesia on a heat pad.

### 4.5. Immunofluorescence Staining

Mice were perfused with phosphate-buffered saline (PBS) followed by 4% paraformaldehyde. Brains were removed, post-fixed, and cryoprotected in 30% sucrose. Coronal sections (20 μm) were cut on a cryostat and processed for immunofluorescence staining. Sections were incubated with primary antibodies against TCF7L2 (Proteintech, Chicago, IL, USA, 13838-1-AP, rabbit polyclonal, 1:100), NeuN (Proteintech, 66836-1-Ig, mouse monoclonal, 1:500) as a neuronal marker and GFP (abcam, Cambridge, UK, ab13970, chicken polyclonal, 1:500), followed by appropriate secondary antibodies. Images were captured using a confocal laser scanning microscope (Zeiss LSM900, Jena, Germany) [52].

### 4.6. Medication Injection

To examine the role of NMDARs, mice were randomly assigned to eight groups: four for NMDA injection (AAV-sh-scrambled + Saline, AAV-sh-scrambled + NMDA, AAV-sh-TCF7L2 + Saline, and AAV-sh-TCF7L2 + NMDA) and four for Memantine injection (AAV-EGFP + Saline, AAV-EGFP + Memantine, AAV-TCF7L2 + Saline, and AAV-TCF7L2 + Memantine). The mice in these groups received intraperitoneal injections of the NMDAR agonist NMDA (75 mg/kg, MCE, HY-17551), or the NMDAR antagonist memantine (15 mg/kg, MCE, HY-B0365A), or saline as a solvent. Behavioral tests (TST and FST) were conducted 30 min after NMDA injection or 2 weeks after memantine injection to assess the effects of NMDAR modulation on depressive-like behaviors in mice with altered TCF7L2 expression.

### 4.7. Statistical Analysis

Data were analyzed using GraphPad Prism 8.0. All data underwent a normal distribution test before the comparisons. An independent-sample *T*-test when normality is satisfied was employed for comparisons between two groups. Otherwise, the Mann–Whitney U-test was performed. Behavioral data were compared using a *T*-test between two groups or one-way analysis of variance (ANOVA) among four groups. After performing ANOVA, the means of each of the two groups were compared using a post hoc least significant difference (LSD) test. The results were presented as mean ± standard error of the mean (SEM), and statistical significance was set at *p* < 0.05, 0.05 < *p* < 0.1 was regarded as a trend.

## Data Availability

Data will be made available on request.
